# Game changer in the diagnosis of bipolar disorder using RNA editing-based blood biomarkers

**DOI:** 10.1192/j.eurpsy.2021.235

**Published:** 2021-08-13

**Authors:** J.-D. Abraham, N. Salvetat, F. Checa-Robles, V. Patel, C. Cayzac, B. Dubuc, D. Vetter, J.-P. Lang, P. Courtet, D. Kupfer, D. Weissmann

**Affiliations:** 1 Neurology, ALCEDIAG/SYS2DIAG, MONTPELLIER, France; 2 Center For Psychiatry And Psychotherapy, Les Toises, Lausanne, Switzerland; 3 Emergency Psychiatry And Acute Care, CHU Montpellier / INSERM, Montpellier, France; 4 Department Of Psychiatry, University of Pittsburgh School of Medicine, Pittsburgh, United States of America

**Keywords:** RNA editing, Blood biomarker, diagnosis, bipolar disorder

## Abstract

**Introduction:**

In clinical practice, differentiating Bipolar Disorder (BD) from unipolar depression is challenging due to the depressive symptoms, which are the core presentations of both disorders. Patients with BD are often misdiagnosed during depressive episodes resulting in a delay in proper treatment and a poor management of their condition.

**Objectives:**

The aim of the present study is to discriminate between unipolar depression and BD using a panel of RNA edited blood biomarkers.

**Methods:**

Depressed patients were classified according to clinical scores in MADRS and IDSC-30 depression scales. After blood collection and RNA extraction, we used whole-transcriptome sequencing to identify differential A-to-I editing events, and Targeted Next Generation Sequencing to validate those biomarkers.

**Results:**

We discovered 646 variants differentially edited between depressed patients and control in a discovery cohort of 57 participants. After using stringent criteria and biological pathway analysis, 6 biomarker candidates were singled out and tested in a validation cohort of 160 patients suffering from unipolar depression and 95 BD patients in a depressive episode, which allowed a differential diagnosis of BD with an AUC of 0.935 and high specificity (Sp=84.6%) and sensitivity (Se=90.9%).

**Conclusions:**

We have shown that a combination of 6 blood RNA editing-related biomarkers allows to discriminate unipolar and bipolar depression This 6 BMKs panel may be crucial to improve BD diagnosis and orientate the treatment therefore addressing the needs of millions of patients suffering from misdiagnosis and incorrect treatment for their diseases. This will change the game for the management of patients.

**Disclosure:**

No significant relationships.

